# Circular dichroism of chiral Majorana states

**DOI:** 10.3762/bjnano.9.110

**Published:** 2018-04-16

**Authors:** Javier Osca, Llorenç Serra

**Affiliations:** 1 Institut de Física Interdisciplinària i de Sistemes Complexos IFISC (CSIC-UIB), E-07122 Palma de Mallorca, Spain; 2 Departament de Física, Universitat de les Illes Balears, E-07122 Palma de Mallorca, Spain

**Keywords:** chiral states, circular dichroism, Majorana modes, optical absorption, topological matter

## Abstract

**Background:** Majorana states in condensed matter devices may be of a localized nature, such as in hybrid semiconductor/superconductor nanowires, or chirally propagating along the edges such as in hybrid 2D quantum-anomalous Hall/superconductor structures.

**Results:** We calculate the circular dichroism due to chiral Majorana states in a hybrid structure made of a quantum-anomalous Hall insulator and a superconductor. The optical absorption of chiral Majorana states is characterized by equally spaced absorption peaks of both positive and negative dichroism. In the limit of a very long structure (a 2D ribbon) peaks of a single sign are favored.

**Conclusion:** Circular-dichroism spectroscopy of chiral Majorana states is suggested as a relevant probe for these peculiar states of topological matter.

## Introduction

The physics of Majorana states in condensed matter devices is attracting strong interest for a few years now [1–8]. The measured zero-bias conductance peaks in hybrid semiconductor/superconductor nanowires have been attributed to the presence of localized Majorana modes on the two ends of the nanowires [9–14]. A Majorana mode enhances the zero-bias conductance by allowing a perfect Andreev backscattering at zero excitation energy when the nanowire is attached to a normal lead. These peculiar pairs of states may be seen as nonlocal split fermions, protected by an energy gap that separates them from other normal states lying at finite energies. Besides the zero energy of the Majorana state, also the conductance peak height was recently seen to coincide with the expected value 2*e*^2^/*h* [15].

Majorana end states in (quasi) 1D nanowires are inherently localized, i.e., their wave function decays exponentially with the distance to the nanowire end. By contrast, propagating Majorana states with sustained spatial oscillations can be present at the edges and along the perimeter of 2D-like hybrid structures. This is the situation in presence of *p* + i*p* superconductivity for spinless quasiparticles, a class of hybrid systems where Majorana states appear around 2D vortex cores in the bulk and on the external edges of the sample [16]. Another class of 2D materials with propagating Majorana modes are the topological insulators based on the quantum-anomalous Hall effect. We refer, specifically, to the hybrid devices of [17], consisting of a quantum-anomalous Hall insulator and a superconductor material. In such systems, chiral Majorana modes propagating along the edges in a clockwise or anticlockwise manner, depending on the orientation of a perpendicular magnetic field, are formed at the 2D interfaces between the quantum-anomalous Hall and the superconductor materials [18–22]. Each chiral Majorana state contributes 0.5*e*^2^/*h* to the linear conductance of the device, such that by tuning the number of Majorana states the conductance takes values 0.5*e*^2^/*h* and 1*e*^2^/*h* for the topological phases with one and two chiral Majorana states, respectively. It is remarkable that the intrinsic magnetization of the material in the anomalous Hall effect allows for the tuning of the phase transitions using much weaker magnetic fields than with the standard Hall effect.

In this work we discuss the connection between chiral Majorana states and optical absorption. We expect that in presence of chiral Majorana states, the optical absorption of circularly polarized light will differ for clockwise and anti-clockwise polarizations. The difference, known as circular dichroism (CD) [23–24], can thus be seen as a measure of the existence of such chiral states. We want to investigate how this behavior is actually realized by explicit calculations of the optical aborption. In previous works we analyzed the optical absorption of localized Majorana states in nanowires [25–26]. In those systems the CD vanishes and the presence of the Majorana state is signaled by a plateau with lower absorption, starting at mid-gap energy, of the *y*-polarized signal with respect to the *x*-polarized signal. It is also worth mentioning that alternative techniques for detecting Majorana fermions, based on microwave photoassisted tunneling in Majorana nanocircuits have been suggested in [27].

For chiral Majorana states in a 2D square or rectangular geometry the CD at low energies is characterized by a sequence of equally spaced peaks, corresponding to transitions of Bogoliubov–deGennes quasiparticles from negative to positive energy. In the usual energy ordering of quasiparticle states (*n* = ±1, ±2, …), the selection rules are: a) transitions between conjugate states *−n*→*n* are forbidden by electron–hole symmetry, b) transitions *−n*→*m* are allowed only when *n* and *m* are both even or both odd. The rationale behind rule b) is the constructive interference of the corresponding quasiparticle states connected by the excitation operator on the edges of the system. Furthermore, it will be shown below that the CD peaks corresponding to those even–even or odd–odd quasiparticle transitions may be either positive or negative. In the limit of a long 2D ribbon there is a preferred CD sign, depending on the magnetic field orientation. For a disc geometry the generalized angular momentum *J_z_* becomes a good quantum number. Then, the combination of circular and particle–hole symmetries in a disc causes a vanishing absorption for *p_x_* ± i*p_y_* fields and, obviously, also a vanishing CD.

## Model

We use the model of [17] for a quantum-anomalous Hall (3D) thin film in contact with two different superconductors. This model represents the device as two surfaces with a certain interaction between them, with Majorana states being located at their edges. In a Nambu spinorial representation that groups the field operators in the top (*t*) and bottom (*b*) layers,


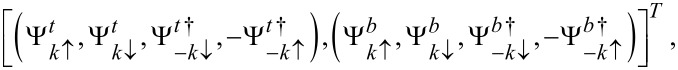


the Hamiltonian is reformulated in the notation of Pauli matrices (with *t* and *b* surfaces corresponding to the Pauli indices 1 and 2, respectively):

[1]
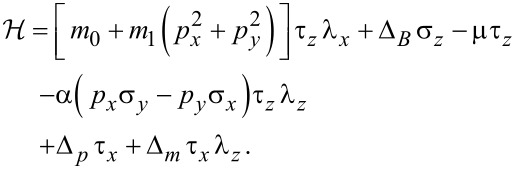


This Hamiltonian is acting in the combined position–spin–isospin–pseudospin space. Spatial positions are treated as a 2D continuum (
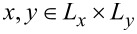
) and a discrete two-valued pseudospin (*z*). The two-valued spin, isospin and pseudospin degrees of freedom are represented by σ, τ and λ Pauli matrices, respectively. As mentioned, the pseudospin (λ) is modeling a coupled bilayer system in which quasiparticles move. The set of Hamiltonian parameters is *m*_0_, *m*_1_, Δ*_B_*, μ, α, Δ*_p_* and Δ*_m_*. The latter two are given in terms of the pairing interaction in the two layers, Δ*_t_* and Δ*_b_*, by

[2]
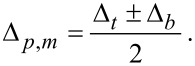


Hybridization of the two surfaces is represented by parameters *m*_0_ and *m*_1_. Δ*_B_* is an effective Zeeman-like parameter including the exchange field associated with the intrinsic magnetization of the material. The chemical potential is given by μ while α represents a Rashba-type spin–orbit interaction.

Below we numerically determine the eigenvalues and eigenstates of 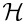 using a 2D grid for *x* and *y*. When Δ*_B_* is increased, the spectrum of low-energy eigenvalues evolves from a gapped (void) spectrum around zero energy at low values of Δ*_B_*, to the emergence of chiral near-zero-energy modes for sufficiently large values of Δ*_B_*. When the pairing parameters for each layer are equal (Δ*_m_* = 0) chiral Majorana states appear in pairs (0–2–…), while for sufficiently different parameters it is Δ*_m_* ≠ 0 and there may be phases with odd numbers of chiral Majorana states as well.

The numerical results shown below are given in an effective unit system, characterized by the choice of 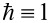, mass *m* ≡ 1/2*m*_1_ ≡ 1 and a chosen length unit *L*_U_, typically *L*_U_ ≈ 1 μm. The corresponding energy unit is then 
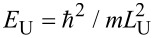
.

### Circular dichroism

We compute the optical absorption cross section for right (+) and left (−) circularly-polarized light from

[3]
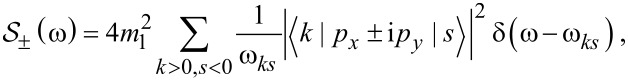


where 
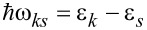
 is the energy difference between particle (unoccupied) and hole (occupied) states. The prefactor 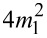 gives the squared inverse effective mass (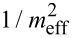) of the Hamiltonian and fixes the dimensions of 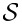 as an area. The circular dichroism at a given frequency 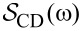 is then defined as the difference between the absorptions for the two circular polarizations,

[4]
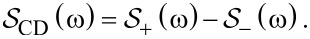


Obviously, in absence of any chirality preference 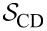 exactly vanishes.

## Results and Discussion

### Chiral bands

**Figure 1:**
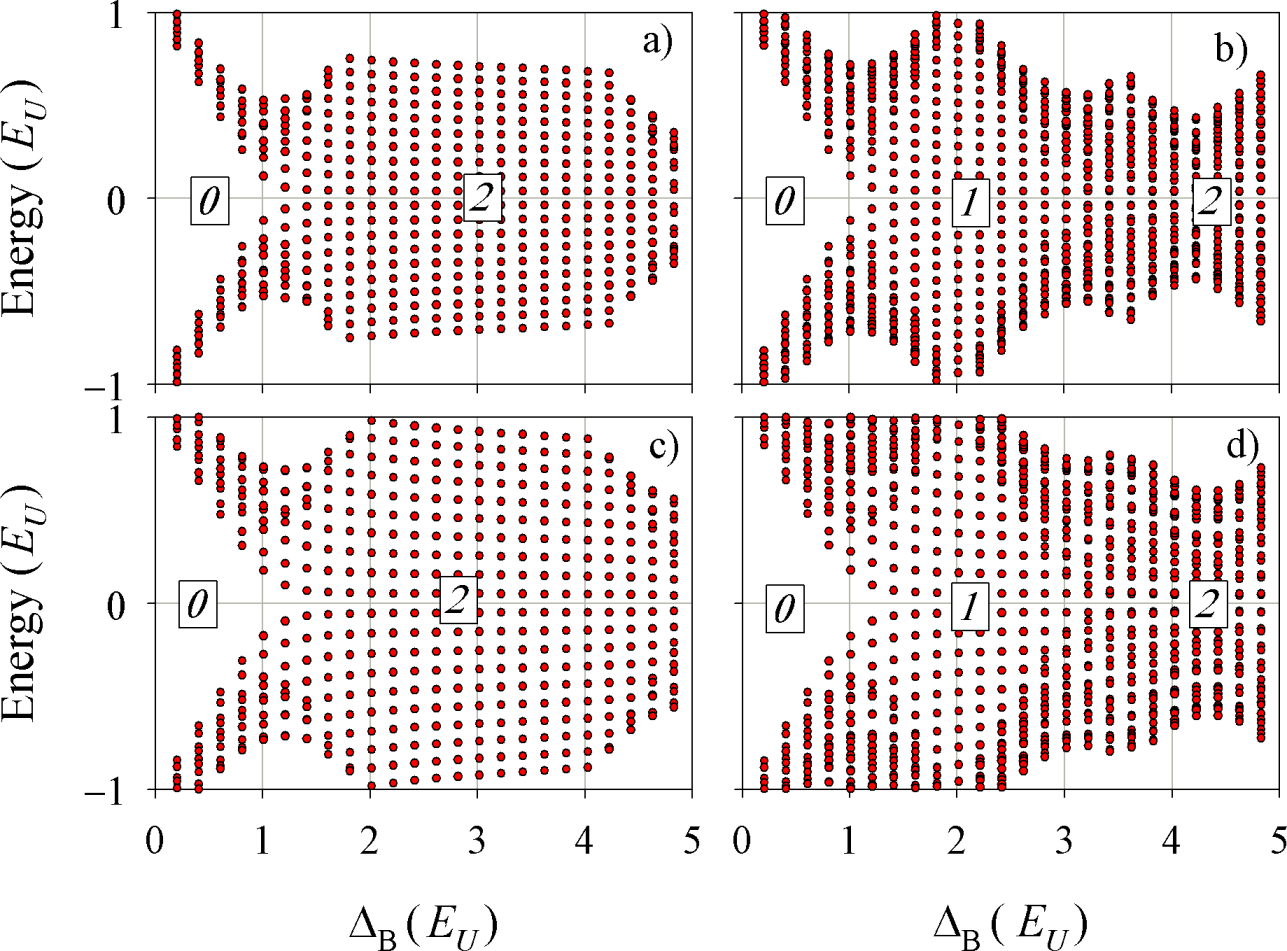
Energy eigenvalues close to zero energy as a function of Δ*_B_*. Panels a) and b) are for a square of dimensions *L_x_* = *L_y_* = 10*L*_U_, while c) and d) correspond to a rectangle of *L_x_* = 2*L_y_* = 20*L*_U_. In a) and c) the same pairing energy is assumed in each layer Δ*_t_* = Δ*_b_* = *E*_U_ while in b) and d) it is Δ*_t_* = Δ*_b_*/3 = *E*_U_. The framed labels indicate the degeneracy of the near-zero energy states, which indicates the topological phase. Other parameters: *m*_0_ = 0, μ = 0, α = *E*_U_*L*_U_.

Figure 1 shows the evolution of the eigenvalue spectrum as a function of the magnetic field parameter Δ*_B_*. The results reproduce already known results [17]. At vanishing Δ*_B_* the spectrum around zero energy is gapped, a gap that tends to close with increasing Δ*_B_* by the appearance of a quasi-continuum distribution of eigenvalues. These low-energy states are indicating the presence of propagating Majorana states, energy-discretized due to the finite size of the system. When Δ*_t_* = Δ*_b_* (Figure 1a,c) the degeneracy is such that the Majorana branches appear in pairs. Directly determining the degeneracy of the energy eigenstates close to zero energy is an alternative way to characterize the topological invariant or Chern number discussed in [20]. We also notice that there is no qualitative difference in the eigenvalue distribution between a square and a rectangle (upper vs lower panels). It is remarkable that when a Majorana phase is well developed the low-energy states are equally spaced in energy. This is particularly clear for 2 *<* Δ*_B_*/*E*_U_ *<* 4 in Figure 1a and Figure 1c, corresponding to the phases with two Majorana states. It can also be seen in Figure 1b and Figure 1d for the phases with one Majorana state while that the equally spaced distribution also hints to the beginning of the phase with two Majorana states.

**Figure 2:**
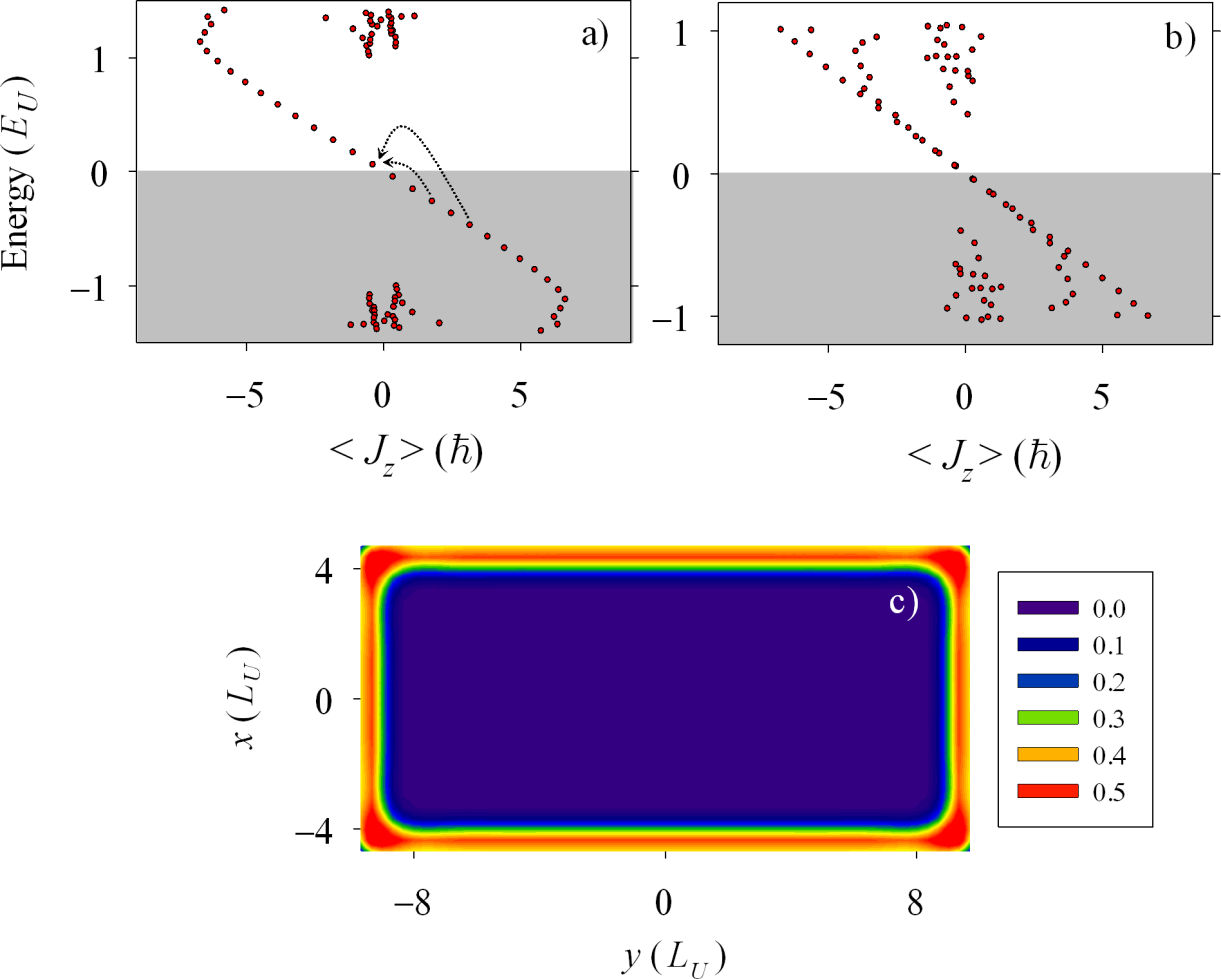
Energy eigenvalues as a function of 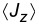. Panels a) and b) correspond to the phases in Figure 1d with one (Δ*_B_* = 2*E*_U_) and two (Δ*_B_* =4.75*E*_U_) Majorana states, respectively. The grey shaded zones indicate the occupied (hole) states while the arrows in panel a) show the two lowest allowed transitions to the first particle state. Panel c) shows the probability density corresponding to the lowest positive-energy state in panel a), adding all spin, isospin and pseudospin contributions.

The chiral character of the gap-closing Majorana states is clearly seen in Figure 2. The equally spaced states at low energy arrange themselves on a line (a chiral band) when plotted as a function of the *z*-component of the angular momentum. For positive Δ*_B_* the angular momentum decreases with increasing energy, causing empty (particle) states to have negative values of 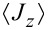, while occupied (hole) states have positive values of 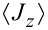. The results of Figure 2a,b correspond to the rectangle with different pairing energies in each layer shown in Figure 1d. For Δ*_B_* = 2*E*_U_ (Figure 2a) there is a single chiral band, while for Δ*_B_* = 4.75*E*_U_ (Figure 2b) there are two overlapping bands. Notice that the overlap of states in Figure 2b degrades as the energy deviates from zero, indicating that the second Majorana band is not yet fully settled for this particular Δ*_B_*. Additionally, Figure 2c explicitly shows the edge character of the states of a chiral Majorana band. A similar distribution is obtained for all the states in a chiral band. On the contrary, states that are not aligned along the chiral band in Figure 2a,b are bulk states separated by a gap from zero energy.

### Absorption and CD

**Figure 3:**
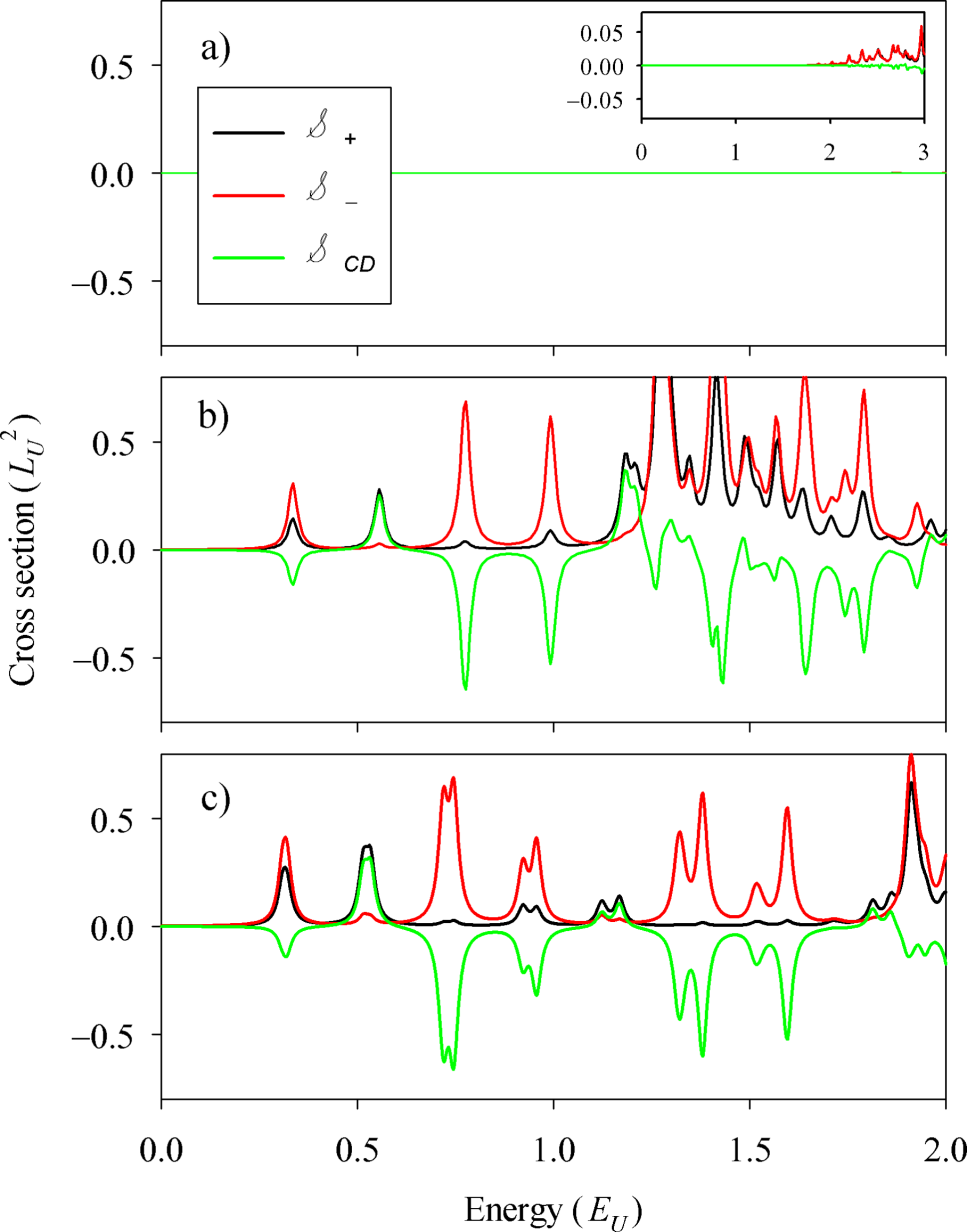
Absorption cross-sections 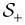, 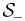 and 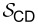 defined in the main text. The shown results correspond to the spectra of Figure 1d for Zeeman parameters of (a) Δ*_B_* = 0.3*E*_U_, (b) 2*E*_U_), and (c) 4.75*E*_U_. The rightmost inset in Figure 3a corresponds to an extended energy range and a zoomed vertical scale for the data of this panel.

Absorption cross-sections and CD for the spectra of the rectangle with different pairing energies in the two layers (Figure 1d) are shown in Figure 3 for selected values of Δ*_B_*. They correspond to zero (Figure 3a), one (Figure 3b) and two (Figure 3c) chiral bands. As anticipated, in presence of the chiral states the system develops a clear CD. For the sake of a better comparison, identical scales have been used in the three panels of Figure 3. In these scales, the two absorptions and the CD essentially vanish in the absence of chiral modes (Figure 3a). The rightmost inset in panel Figure 3a shows that for energies exceeding the quasiparticle gap a small absorption eventually appears due to transitions between bulk states (cf. Figure 1d). However, the CD still vanishes within numerical precision. The negative CD peaks dominate in Figure 3b,c due to the negative slope of the chiral bands (Figure 2a,b). It is remarkable, however, that a few positive peaks are also present. We attribute them to the fact that in a rectangular geometry *J_z_* is not a good quantum number and, therefore, there are states with mixed angular momentum. We have performed calculations in a circular geometry confirming this interpretation. Therefore, quasiparticle scattering by the corners plays a nontrivial role on the absorption by chiral edge states.

The most conspicuous feature of Figure 3b is the regular energy spacing of the first few CD peaks. Analysing them in terms of energy transitions of the chiral band it is easily noticed that they correspond to jumps of 3, 5, 7,… steps (see arrows in Figure 2a). We explain this selection rule noticing the following restrictions for transitions from the negative *n*-th state to the positive *m*-th state (*−n*→*m*): (a) Transitions between conjugate states *−n*→*n* are forbidden by particle–hole symmetry [25], and (b) *n* even to *m* odd transitions (or vice versa) are forbidden because of destructive interference along the nanostructure perimeter with the excitation operator. This rule is far less obvious than rule (a) and results from the approximately 1D character of the chiral edge modes and the interference induced by the propagation through corners. Indeed, we have seen that for active transitions within the chiral bands the regions around the corners are those contributing the most to the matrix element in Equation 3.

For a disc, *J_z_* becomes a good symmetry and, by angular momentum conservation with a dipole operator only the transition −1→1 is possible. However, this transition is blocked by rule (a) and, therefore, no dipole absorption is possible and the CD exactly vanishes. We have also checked this behavior by explicit calculation for a device with circular geometry. For a square and rectangle, quasiparticle scattering by the corners plays a nontrivial role yielding the mentioned deviations with respect to the disc.

The pattern of equally spaced peaks is fulfilled only when one or several chiral bands are fully developed and they exactly overlap. In Figure 3c we see that the slight degradation of the two-band overlaps of Figure 2b manifests in a small twofold splitting of the CD peaks. It is also worth stressing that once the chiral bands are fully formed, the energy positions of the first few CD peaks become independent on Δ*_B_* (cf. Figure 2b and Figure 2c).

**Figure 4:**
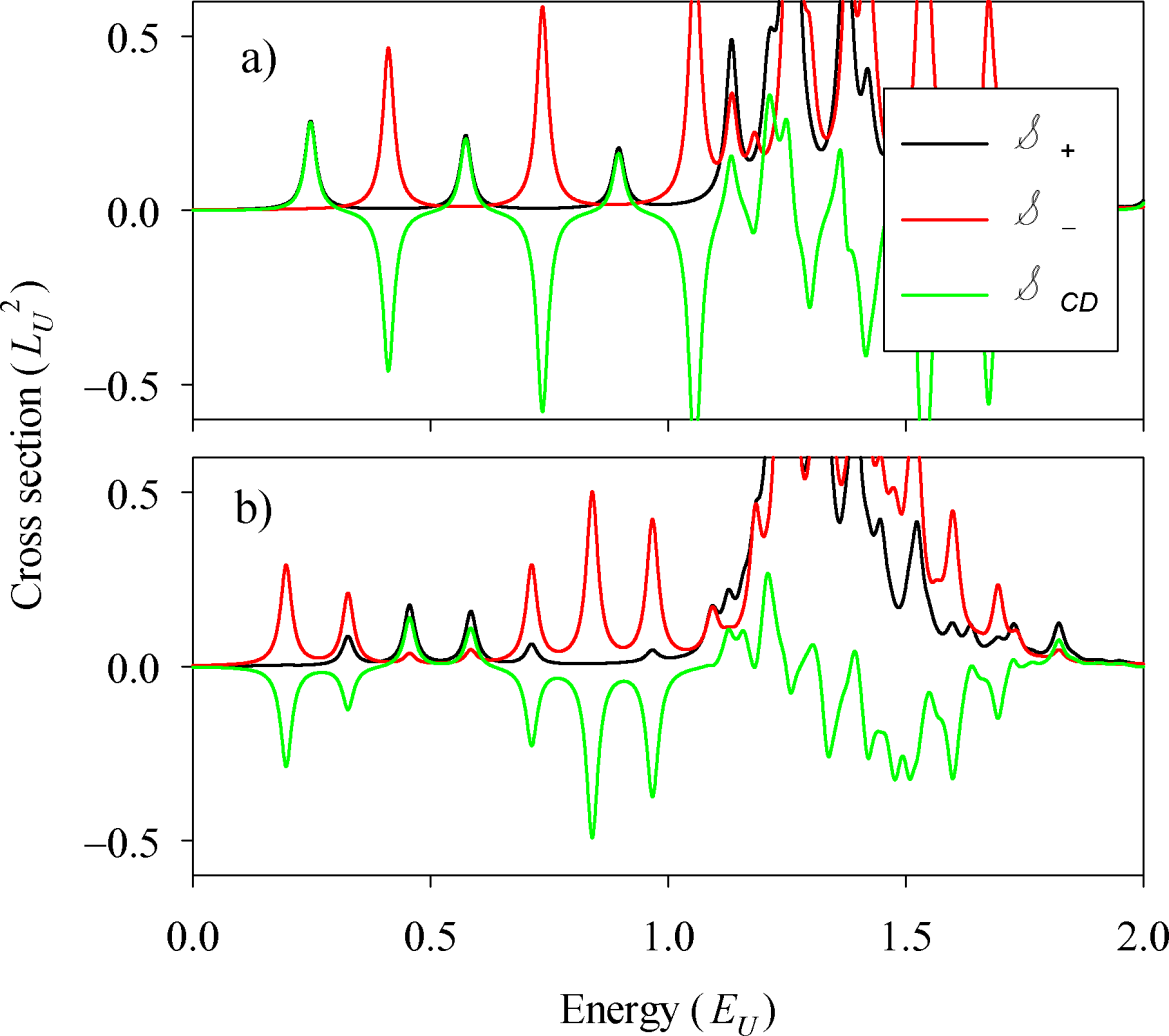
Absorption cross-sections 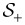, 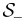 and 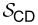 for (a) a square of *L_x_* = *L_y_* = 20 *L*_U_), and for (b) a rectangle of *L_x_* = 6*L_y_* = 60*L*_U_ (b). In both cases we used Δ*_B_* = 2*E*_U_ and Δ*_t_* = Δ*_b_*/3 = *E*_U_.

Figure 4 shows the absorption results for different geometries, a square (Figure 4a) and a long rectangle resembling a 2D ribbon (Figure 4b). For the square, the first CD peaks alter sign in a remarkable way. For the ribbon the alternation is of a longer period, the positive peaks having a much lower intensity than the negative ones and there are groups of a few consecutive negative peaks. The 2D ribbon shape thus favors the observation of CD peaks of the same sign. Nevertheless, the presence of the corners is still essential since for a strictly infinite ribbon the CD exactly vanishes. This is clear when realizing that with fully translational invariant states the *p_x_* operator in Equation 3 is not yielding any excitation and, therefore, the sign of the *p_y_* operator becomes irrelevant, yielding 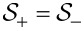.

## Conclusion

In this work we have investigated the manifestation of chiral Majorana modes in the CD of the dipole absorption. The chiral bands formed at the edges of a hybrid system made of a quantum-anomalous Hall insulator and a superconductor yield equally spaced peaks in the CD signal. We identified the particle–hole selection rules responsible for this behavior from the analysis in terms of chiral bands. In a disc there is no CD signal due to the incompatibility of the selection rules with the angular momentum restriction; a square or rectangular geometry (or, more generally, a system with straight edges or breaking circular symmetry) is needed. The presence of two chiral bands can be inferred from the small splitting of the CD peaks. Finally, both positive and negative CD peaks can be seen, with a perfect alternation in a square and a favored sign in a long 2D ribbon geometry.

Our results suggest the use of CD spectroscopy as a valuable probe of chiral Majorana states, complementing the evidences obtained with electrical conductance measurements [17]. This may require the use of an array of absorbing devices, in order to achieve a combined signal of sufficient intensity. Alternatively, techniques such as those developed for single plasmonic nanoparticle sensing [28] might be applied to an isolated chiral Majorana device. Particularly, among the latter we stress the techniques for single-particle absorption that have allowed measuring the extinction spectrum of a single silica shell-coated silver nanoparticle excited with varying polarizations [29].
